# Comparison of Digital Versus Conventional Documentation of Ward Round in Terms of Staff Satisfaction, Effect on Education, and Adherence to British Orthopaedic Association Guidelines

**DOI:** 10.7759/cureus.27598

**Published:** 2022-08-02

**Authors:** Muhammad I Chaudary, Junaid Zeb, Faizan Arshad, Salman Sadiq, Umar-Khetaab Hanif, Usman Saleem, Fouad Chaudhry

**Affiliations:** 1 Trauma and Orthopaedics, Russells Hall Hospital, Dudley, GBR; 2 Trauma and Orthopaedics, University Hospitals Coventry and Warwickshire, Coventry, GBR; 3 Trauma and Orthopaedics, The Royal Wolverhampton NHS Trust, Wolverhampton, GBR

**Keywords:** trauma, orthopaedics, staff satisfaction, boa guidelines, paperwork, electronic record

## Abstract

Objective

To compare the role of paper-based versus digital record keeping in the orthopaedic ward in terms of staff satisfaction, education of staff, and adherence to British Orthopaedic Association (BOA) guidelines.

Materials and methods

Forty-four participants including nurses, senior house officers, foundation year trainees, and consultants completed a questionnaire. The first survey was done to introduce electronic records keeping to the participants and the second survey was conducted to review the collected record. Three parameters were assessed, which were adherence to BOA guidelines, staff satisfaction, and effect of education for both paper-based and electronic records. Comparison between two methods of record keeping was done by independent t-test for continuous data and chi-square test for categorical.

Results

For all four questions about staff satisfaction, the score of the electronic method was higher than paperwork statistically. The score for ‘opportunity to learn images in ward round’ was higher in electronic (3.9±0.8) than paperwork (2.6±1.3) statistically (p<0.001). Comparable results were found for ‘educational usefulness of ward round’ and ‘typing time affecting learning time’. For adherence to guidelines, the electronic record keeping was more effective in storing the patient’s ID and name (p=0.05), details of documenting clinician (p<0.001), time of ward round ((p=0.005), whom to contact in case of concern (p=0.050), and grade of ward round clinician (<0.001).

Conclusion

Electronic records in the orthopaedic ward were deemed better than paperwork in terms of staff satisfaction, positive effect on the education of doctors, and adherence to BOA guidelines.

## Introduction

Proper documentation in patient records is essential for the patient and clinician [1]. These records can be used for audit, research, and dealing with medicolegal issues. The Royal College of Surgeons of England states that medical records are 'fundamental for clinical care and audit of surgical services' and the British Orthopedic Association (BOA) tell us 'good records are a basic tool of clinical practice', emphasizing the role of record-keeping [2].

Traditionally, health records were collected on paper, stored in folders categorized into sections based on the type of note, and a single set was available [3]. Due to the availability of new computer technology, electronic health record (EHR) is now readily available [4]. The EHRs have made it feasible to record patients' medical information for reading availability in cloud form, to change the health records format, and ultimately the health care [5].

Electronic records are associated with job satisfaction among health workers and physicians due to reproducibility, easy handling, ability to share with others, no space issues, and easy manipulation [6,7]. In most practices, dual record documentation is followed both on paper and computer for each patient. The hospital administration needs to decide to use either of the methods and build up the health workers' ability for the chosen method for maintenance of quality records [8].

The BOA guidelines were introduced by The Royal College of Physicians (RCP), London, United Kingdom, for better patient care based on evidence-based research and updated with time. The clinical audit usually involves how much the existing practice in an institute or private practice follows the BOA guidelines [9,10].

The record-keeping aims are to provide better patient care, offer data to audit, educate young doctors, handle medico-legal issues, perform research, and follow the standard guidelines of care. This study was conducted to compare the role of paper-based versus digital record keeping in the orthopaedic ward in terms of adherence to BOA guidelines, staff satisfaction, and education. To our knowledge, there is no previous study on this perspective.

## Materials and methods

This comparative study was conducted at the Department of Trauma & Orthopaedics at Russells Hall Hospital, Dudley, United Kingdom, on 44 participants including nurses, senior house officers, foundation year trainees, and consultants from February 2021 to August 2021 in the form of two surveys. The first survey was done in February 2021 to introduce electronic records keeping to the staff and the second survey was conducted in August 2021 to review the collected records.

Three parameters were assessed, which were adherence to BOA guidelines, staff satisfaction, and the effect of education for both paper-based and electronic records. Adherence to BOA guidelines was assessed in the first loop (survey) review of all inpatient's paper notes, and ward round entries and comparing them against BOA guidelines while the same was done in the second survey for electronic records. The parameters assessed in 'adherence to BOA guidelines' were the efficacy of paper-based or electronic method to record the patient's ID, patient's name, name of the clinician in ward round, details of clinicians, grade of ward round clinician, date and time of ward round, and contact details of the clinician in case of any concern.

Staff satisfaction was assessed on a 5-point Likert scale from 1 (extremely dissatisfied) to 5 (extremely satisfied) as a snapshot survey at any one-time point. For recording the effect of education, four questions were asked in a staff survey enquiring juniors (house officers and registrars) if they found ward round an educationally enriching experience, comparing paper versus electronic methods.

Data analysis was done in IBM SPSS Statistics for Windows, Version 22.0 (Released 2013; IBM Corp., Armonk, New York, United States). Mean with SD was calculated for continuous data like Likert score and frequencies along with percentages for categorical data. Comparison between two methods of record keeping was done by independent t-test for continuous data and chi-square test for categorical. In case of violation of chi-square assumption, Fisher's exact was run. P≤0.05 was kept as the level of significance.

## Results

The highest number of participants were nurses (n=14, 31.82%) and senior house officers/foundation years/physician assistants (n=13, 29.5%) followed by registrars (n=8, 18.18%) and consultants (n=5, 11.36%) (Table 1).

**Table 1:** Frequency of participants SHO: senior house officer; FY: foundation years; PA; physician associate

Participants	n (%)
Nurses	14 (31.82)
SHOs/FY/PA	13 (29.5)
Registrars	8 (18.18)
Consultant	5 (11.36)
Others (clinical support workers)	4 (9.09)
Total	44

A questionnaire was used to assess staff satisfaction with both paperwork and electronic documentation. The staff satisfaction on the Likert scale about 'confidence about clarity of treatment plan' was significantly (p=0.003) higher in electronic (4.2± 0.9) than paperwork (3.5±1.32). Similarly, the Likert score for 'name of ward round clinician', 'documenting clinician', 'date of ward round', 'time of ward round', and 'whom to contact' was very higher statistically (p<0.001) in the electronic method than paperwork (Table 2).

**Table 2:** Comparison of staff satisfaction score and effect on education in paperwork versus electronic documentation *Independent t-test

Variables	Paperwork	Electronic	P-Value*
	Mean Score ± SD	Mean Score ± SD	
Confidence in clarity of treatment plan	3.5±1.32	4.2± 0.9	0.003
Ward round clinician name	3.9±1	4.6±0.6	<0.001
Documenting clinician	2.8±1.2	4.5±0.7	<0.001
Date of Ward round	4±0.8	4.9±0.3	<0.001
Time of ward round	3.3±1	4.7±0.7	<0.001
Whom to contact	2.8±1.2	3.8±1	<0.001
Opportunity to review images in ward round	3.4±1	4±0.7	0.001
Opportunity to learn images in ward round	2.6±1.3	3.9±0.8	<0.001
Confidence to review images with seniors	3.2±1.3	3.7±1.2	0.11
Educational usefulness of ward round	2.1±1	3.7±1.1	<0.001
Typing time affects learning time	2.8±1	3.8±1.2	0.01

Four questions were asked about the effect on staff education. The score for 'opportunity to learn images in ward round' was higher in electronic (3.9±0.8) than paperwork (2.6±1.3) statistically (p<0.001). Comparable results were found for 'educational usefulness of ward round' and 'typing time affecting learning time'. However, though the score for 'confidence to review images with seniors' was a little higher in the electronic method (3.7±1.2) than paperwork (3.2±1.3), it was not statistically significant (p=0.11) (Table 2).

For adherence to BOA guidelines, electronic record keeping was more effective in storing in patient's ID and name (p=0.05), details of documenting clinician (p<0.001), time of ward round (p=0.005), whom to contact in case of concern (p=0.05), and grade of ward round clinician (<0.001). However, the difference was not statistically significant for ward round clinicians (p=0.41) and the date of ward round (p=.21) (Table 3).

**Table 3:** Comparison of adherence to guidelines in paperwork versus electronic documentation

Variables	Paperwork n (%)		Electronic n (%)		P-Values
Patient identification and name	Yes	29 (65.91)	Yes	34 (100)	0.05
	No	5 (11.36)	No	0	
Ward round clinician	Yes	32 (72.73)	Yes	34 (100)	0.41
	No	2 (4.545)	No	0 (0)	
Documenting clinician	Yes	17 (38.64)	Yes	34 (100)	<0.001
	No	17 (38.64)	No	0 (0)	
Date of the ward round	Yes	31 (70.45)	Yes	34 (100)	0.21
	No	3 (6.82)	No	0 (0)	
Time of ward round	Yes	26 (59.09)	Yes	34 (100)	0.005
	No	8 (18.18)	No	0 (0)	
Whom to contact in case of a concern	Yes	0 (0)	Yes	5 (14.70)	0.05
	No	34 (77.27)	No	29 (85.29)	
Grade of ward round clinician	Yes	18 (40.91)	Yes	34 (100)	<0.001
	No	16 (36.36)	No	0 (0)	

## Discussion

This study was conducted to know whether electronic record-keeping is better than paperwork in terms of staff satisfaction, the effect on the education of junior doctors, and adherence to BOA guidelines. This study suggests that electronic health records are better in terms of the above-mentioned parameters.

We found that staff satisfaction was higher for 'confidence on clarity of treatment plan' in electronic than in paperwork. The treatment plan is the road map by which care is provided to the patient in a customised and orderly way [11]. Proper documentation of the treatment plan cannot be underestimated. A well-documented treatment plan helps the multiple consultants and nurses to care for the patients in a unified and streamlined way without confusion [12]. Paper records sometimes are of inadequate quality due to poor handwriting, not enough details due to lack of time, and issues with storage of hard copies [13]. Electronic records are much easier to collect, modify, share, store, and reproduce [14].

The results of this study showed that staff satisfaction was higher from electronic record-keeping than paperwork. In North Carolina, United States, a study was conducted on the association between job satisfaction and electronic record-keeping among primary care physicians. They reported a positive association between job satisfaction and electronic health records [6].

BOA established the guidelines for the management of patients in orthopaedics under the standard protocol. These guidelines have been introduced on evidence-based research. In the orthopaedics ward, trauma is the most frequent type of presenting illness, which requires prompt care to prevent complications. BOA guidelines include patients’ ID and name, ward round clinician, documenting clinician, date of ward round, time of ward round, whom to contact in case of concern, and grade of ward round clinician. These guidelines can be documented by both electronic and paper methods. Hence, by following these guidelines, many surgical and medical complications, as well as medico-legal issues, can be overcome. To adhere to the BOA guidelines, proper documentation of a patient's condition and personal data is of paramount importance [15].

Patient safety is key to good medical practice. In centres with busy trauma and workload, following certain pathways and guidelines are of paramount importance in order to improve the documentation quality, especially in handover and continuity of patient care and hence patients safety [16]. Green et al. reported a significant improvement of compliance of ward round documentation of 97% when they re-audited ward round documentation after the introduction of the electronic notes system (p=0.0012); moreover, they also reported significant improvement in the consistency of ward round entries (p<0.00001) and after third loop, they noticed that this improved level of documentation was maintained although there was no improvement compared to the second cycle (P=0.203) [17]. Our study showed that BOA guidelines can be followed more easily by electronic methods than paper-based and have showed significant improvement in ward round documentation.

The major limitation of this research was the relatively small sample size, which can reduce the power of the study and, hence, the results of statistical inference. This is a single-centre study; multi-centre studies can better explore this area. Moreover, the participants of this study were having different qualification and experience and were not equally distributed. Also, data were collected separately in each cycle and reflected the presence of documentation of individual criteria and were not cross-checked with paper documentation to make sure whether all documented information was correct or not. 

## Conclusions

Within the limitations of this study, it can be concluded that electronic record-keeping in orthopaedic ward rounds is better than paperwork in terms of staff satisfaction, positive effect on the education of junior doctors, and adherence to BOA guidelines. The most common presentation among orthopaedic emergencies is trauma, which requires prompt treatment to avoid long-term complications. By strictly adhering to BOA guidelines for documenting the ward round, including the patient's condition, and treatment plan, many complications, including medico-legal issues, can be greatly avoided.
